# Facial Expression Recognition, Fear Conditioning, and Startle Modulation in Female Subjects with Conduct Disorder

**DOI:** 10.1016/j.biopsych.2010.02.019

**Published:** 2010-08-01

**Authors:** Graeme Fairchild, Yvette Stobbe, Stephanie H.M. van Goozen, Andrew J. Calder, Ian M. Goodyer

**Affiliations:** a Developmental Psychiatry Section, Department of Psychiatry, Cambridge University, Cambridge; b School of Psychology, Cardiff University, Cardiff, Wales; c Medical Research Council Cognition and Brain Sciences Unit, Cambridge, United Kingdom

**Keywords:** Conduct disorder, emotion, face recognition, female, psychopathy

## Abstract

**Background:**

Recent behavioral and psychophysiological studies have provided converging evidence for emotional dysfunction in conduct disorder (CD). Most of these studies focused on male subjects and little is known about emotional processing in female subjects with CD. Our primary aim was to characterize explicit and implicit aspects of emotion function to determine whether deficits in these processes are present in girls with CD.

**Methods:**

Female adolescents with CD (*n* = 25) and control subjects with no history of severe antisocial behavior and no current psychiatric disorder (*n* = 30) completed tasks measuring facial expression and facial identity recognition, differential autonomic conditioning, and affective modulation of the startle reflex by picture valence.

**Results:**

Compared with control subjects, participants with CD showed impaired recognition of anger and disgust but no differences in facial identity recognition. Impaired sadness recognition was observed in CD participants high in psychopathic traits relative to those lower in psychopathic traits. Participants with CD displayed reduced skin conductance responses to an aversive unconditioned stimulus and impaired autonomic discrimination between the conditioned stimuli, indicating impaired fear conditioning. Participants with CD also showed reduced startle magnitudes across picture valence types, but there were no significant group differences in the pattern of affective modulation.

**Conclusions:**

Adolescent female subjects with CD exhibited deficits in explicit and implicit tests of emotion function and reduced autonomic responsiveness across different output systems. There were, however, no differences in emotional reactivity. These findings suggest that emotional recognition and learning are impaired in female subjects with CD, consistent with results previously obtained in male subjects with CD.

Conduct disorder (CD) is a psychiatric diagnosis characterized by increased levels of aggressive and antisocial behavior. Although CD is less common in females than males (1,2), it is nevertheless the second most frequently diagnosed psychiatric disorder in female adolescents (3–5). Conduct disorder in female adolescents is associated with negative psychosocial outcomes including early pregnancy (6) and increased rates of morbidity, substance dependence, criminality, and psychiatric illness in adulthood (7,8).

We and others have argued that deficits in emotional learning and recognition contribute to the etiology of antisocial behavior (9–12). Although there is converging evidence for the presence of such deficits in male adolescents with CD (13,14), little is known about emotional learning, recognition, or reactivity in female adolescents with CD. This study addresses these gaps in the literature.

Facial expressions are an important channel of social communication, carrying information about others' emotional states. Conduct disorder is associated with impaired social functioning that may stem from difficulties in perceiving others' feelings. We therefore assessed our participants' ability to recognize facial expressions. Given previous findings in male subjects (14), we predicted impaired anger and disgust recognition in girls with CD. We also hypothesized that variation in psychopathic traits would influence recognition of fear and sadness (14,15). The Benton Facial Recognition Test (16) assessed for potential group differences in face perception skills.

We studied fear conditioning as an objective measure of emotional learning. Skin conductance responses (SCRs) to conditioned stimuli positive (CS+) were measured before and after the CS+ were paired with an aversive unconditioned stimulus (US) to examine transfer of fear responses from the US to the CS+. Patients with anxiety disorders show enhanced fear conditioning (17), whereas fear-conditioning deficits are observed in adult psychopaths (18–21) and male subjects with CD (13). Consequently, we predicted impaired conditioning in female subjects with CD.

Finally, we assessed affective modulation of the startle reflex, a sensitive index of emotional reactivity (22–26). Eyeblink startle reflexes to an acoustic probe are typically attenuated when pleasant images are viewed and potentiated by negative images (27). This pattern of modulation is proposed to result from priming of appetitive or defensive motivational systems by the visual images (28). Affective startle modulation has been used to investigate emotion-related psychopathology, with anxious individuals showing increased (29) and psychopaths showing reduced (30–32) potentiation by negative images. Previous work in male adolescents with CD or children with oppositional defiant disorder (ODD) did not reveal aberrant patterns of affective modulation, but overall startle magnitudes were reduced in both groups compared with control subjects (13,33). We therefore predicted reduced startle magnitudes but a normal pattern of affective modulation in female subjects with CD.

## Methods and Materials

### Participants

Fifty-five female adolescents aged 14 to 18 years were recruited from schools, pupil referral units, and the Cambridge Youth Offending Service. Twenty-five participants had CD or ODD (three had ODD only). Most (*n* = 22) participants with CD/ODD had adolescence-onset CD/ODD (i.e., onset of symptoms and functional impairment after age 10 [34]). For brevity, the term CD refers to the overall group with disruptive behavior disorders. We also tested 30 age- and sex-matched healthy control subjects (no lifetime CD/ODD and no current psychiatric illness). The Local Research Ethics Committee approved the study and all participants provided written informed consent.

All participants were in midpuberty to postpuberty (Tanner Stage III or above [35]) and were tested 5 to 12 days after the onset of menstruation (early- to mid-follicular phase) because startle magnitudes and facial expression recognition accuracy may vary during the menstrual cycle (36,37).

Exclusion criteria included full-scale IQ <75 as estimated using matrix reasoning and vocabulary subtests of the Wechsler Abbreviated Scale of Intelligence (38), presence of pervasive developmental disorder (e.g., autism) or chronic physical illness, and current steroid medication use.

Participants were assessed for CD, ODD, attention-deficit/hyperactivity disorder (ADHD), major depressive disorder (MDD), generalized anxiety disorder, obsessive compulsive disorder, and posttraumatic stress disorder using the Schedule for Affective Disorders and Schizophrenia for School-Age Children-Present and Lifetime version (39). Separate diagnostic interviews were carried out with participants and their caregivers.

Psychopathic traits were measured using the Youth Psychopathic traits Inventory (YPI) (40). Participants with total scores ≥ 2.5 were classified as being high in psychopathic traits (41). We assessed callous-unemotional traits using the callous-unemotional dimension YPI subscale (40). Anxiety symptoms were measured using the Revised Children's Manifest Anxiety Scale (42). The Adolescent Alcohol and Drug Involvement Scale assessed alcohol and substance use (43). Socioeconomic status was estimated using the occupation of the participant's caregiver using the Standard Occupational Classification 2000 (44).

### Facial Identity Perception

The Benton Facial Recognition Test (16) assesses participants' ability to match pictures of unfamiliar faces and was used to screen for basic face perception deficits. Participants are asked to identify a target face(s) from an array of six faces, presented under different illumination or head orientation conditions.

### Facial Expression Recognition

The emotion hexagon task was used to assess facial expression recognition ability (45). Participants were asked to label morphed facial expression continua spanning the following six expression pairs: happiness-surprise, surprise-fear, fear-sadness, sadness-disgust, disgust-anger, and anger-happiness (Figure S1 in Supplement 1). For example, for happiness-surprise, facial images of happy and surprised expressions were morphed to create a series of pictures ranging across five ratios (90%–10%, 70%–30%, 50%–50%, 30%–70%, and 10%–90%). Morphed faces were presented individually on a computer monitor in random order. Each face was presented for a maximum of 5 sec and participants were asked to label the expression displayed. After a practice block, participants completed five blocks, with each block containing one instance of each of the 20 morphed faces (and four instances of each expression). For each expression, the total score ranged from 0 to 20, with 50%-50% morphed images not being scored.

### Aversive Conditioning

#### Skin Conductance Recording

Electrodermal activity was measured at the distal phalanges of the index and middle fingers of the nondominant hand, using a skin conductance transducer (TSD203) and amplifier (GSR100C) connected to an MP150 system (all BIOPAC Systems, Goleta, California). The electrodes of the transducer were filled with skin conductance paste, and electrodermal activity was sampled at 50 Hz. The task computer sent digital markers to the MP150 system to indicate slide onset and offset and also onset of the white noise tone. Data were analyzed offline using AcqKnowledge 3.8.1 (BIOPAC Systems).

#### Fear Conditioning Procedure

We assessed differential conditioning (CS+ = blue slides and conditioned stimulus negative [CS–] = red slides) with partial (56%) reinforcement during acquisition, following Bechara and Damasio (46). Briefly, we used monochrome slides as the visual conditioned stimuli (CS), a loud (97 dB) aversive white noise tone lasting 1000 msec as the US, and the amplitude of the SCR in the 7-sec period following CS presentation as the dependent measure of the conditioning process. The colored slides were presented for 3 sec with a 10-sec interslide interval. The aversive tone was presented binaurally via headphones. When presented with the CS+ (reinforced trials), the white noise tone occurred 2 sec after slide onset. The conditioned SCR amplitude within the 7-sec analysis window was quantified using the peak-to-peak function in AcqKnowledge and the slope function to determine the direction of the change (positive or negative).

The procedure involved four phases: habituation (HAB), acquisition 1 (ACQ1), acquisition 2 (ACQ2), and extinction (EXT). During the habituation phase, the average SCR elicited by the CS+ was compared with that elicited by the CS– (two of each slide type were presented). In the acquisition phases, the average SCR to the unreinforced CS+ was compared with the average SCR to the CS−. Acquisition 1 comprised the first four unreinforced CS+, five reinforced CS+, and five presentations of the CS–, presented in pseudorandom order. Acquisition 2 involved the same combination of CS+ and CS– slides in a different order. The EXT phase comprised six unreinforced CS+ and three CS– trials. Again, average SCRs to the CS+ and CS– were compared.

After the experiment had finished, participants were asked to recall salient aspects of the task to ensure that they had been paying attention, i.e., how many and which colors they had seen and the number of slides and the color that had been paired with the aversive sound.

### Affective Modulation of the Startle Reflex

We modified the design employed by Patrick *et al.* (30). Participants viewed nine positive, nine neutral, nine sad, nine disgusting, and nine fearful images from the International Affective Pictures System (47) (see [13] for International Affective Pictures System numbers and normative valence and arousal ratings). Slides were presented in a single, fixed, pseudorandom sequence. Each slide was displayed for 10 sec with an interslide interval of 10 sec.

Eyeblink responses to the startle probes were measured using silver/silver chloride electrodes positioned over the orbicularis oculi muscle (48). Electromyographic data were recorded at 1000 Hz using an EMG100C amplifier module (BIOPAC Systems), with a bandpass of 30 Hz to 500 Hz. A 97-dB white noise probe lasting 100 msec was presented binaurally via headphones. To reduce the impact of habituation, only 30 slides were accompanied by the startle probe (6 from each slide category). The slides were presented in blocks to examine for possible effects of habituation. Probe presentation was varied relative to slide onset (3500, 4500, or 5500 msec after onset).

Startle responses were quantified offline using AcqKnowledge functions “Max” and “Min” in the electromyographic channel using an analysis window beginning 30 msec after and terminating 100 msec after startle probe onset. These values were subtracted from baseline Max and Min values within the 50-msec period before probe onset to yield integrated values for startle magnitude.

### Data Analyses

Chi-square and independent *t* tests were used to investigate group differences in demographic and personality characteristics. Independent *t* tests compared groups on the Benton Facial Recognition Test. All other statistical tests are described in the Results section. Where the data violated sphericity, Huynh-Feldt or Greenhouse-Geisser correction was applied as appropriate (49). Effect sizes are reported as *r* equivalent (50) (abbreviated to *r*; small ≥.10, medium ≥.30, large ≥.50; [51]) or partial eta-squared. Analyses were conducted using SPSS 11.5 (SPSS, Inc., Chicago, Illinois).

## Results

### Demographic Characteristics

The groups were matched for age, ethnicity, and socioeconomic status, but control subjects were of higher intelligence than CD participants (*p* < .005; Table 1). Compared with control subjects, significantly more CD participants reported regular use of tobacco [χ^2^(1) = 27.5, *p* < .001], alcohol [χ^2^(1) = 8.5, *p* < .005], and cannabis [χ^2^(1) = 15.5, *p* < .001]. Conduct disorder participants also had higher levels of psychopathic (*p* < .001) and callous-unemotional (*p* < .001) traits. There were no group differences in self-reported anxiety.

### Facial Identity Recognition

There was no group difference in face recognition [*t*(53) = 1.13, *p* = .26]. Mean (± SD) score for control subjects = 46.4/54 (± 2.9) and for CD participants = 45.6/54 (± 2.5).

### Facial Expression Recognition

**Figure 1:**
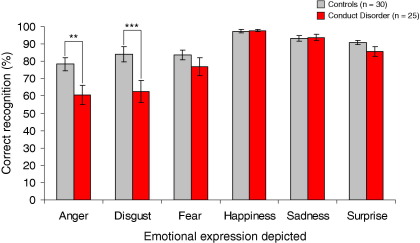
Accuracy of facial expression recognition by group. Relative to control subjects, participants with conduct disorder showed impairments in anger and disgust recognition. **p < .01, ***p < .005.

The emotion hexagon task results are shown in Figure 1. The data were not normally distributed and could not be transformed to a normal distribution. Mann-Whitney tests were therefore used to compare groups on the six expressions separately, using an alpha level of .008 (i.e., .05/6) to control for multiple comparisons.

Relative to control subjects, CD participants showed significant deficits in recognition of anger (*Z* = −2.70, *p* = .007, *r* = .35) and disgust (*Z* = −2.86, *p* = .004, *r* = .37) but not fear, happiness, sadness, or surprise (all *p* > .1).

Confusion matrices showing which emotion labels were selected if the target emotion was misidentified are provided in Table S1A and Table S1B in Supplement 1.

The anger and disgust recognition deficits were still significant (both *p* = .03; *r* = .30 and *r* = .31, respectively) after equating groups on estimated IQ (*p* = .13) by removing eight high IQ control subjects (>118 on the Wechsler Abbreviated Scale of Intelligence). This procedure reduced sample size; thus, an alpha of .05 was used. To rule out the possibility that the group findings were driven by comorbid MDD or ADHD, we omitted participants with these disorders and repeated the analyses. The findings for anger and disgust were significant (both *p* = .01, *r* = .33) when participants with MDD were excluded. Following exclusion of participants with ADHD, the disgust deficit was significant (*p* < .01, *r* = .35) and the anger deficit was of borderline significance (*p* = .055, *r* = .27).

### Effect of Psychopathic Traits on Facial Expression Recognition

**Figure 2:**
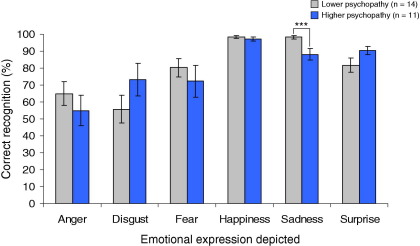
Effect of psychopathic traits on facial expression recognition, considering participants with conduct disorder only. Participants high in psychopathic traits, as measured using total scores on the Youth Psychopathic traits Inventory, showed a specific deficit in recognition of sadness relative to conduct disorder participants lower in psychopathic traits. ***p < .005.

Given our a priori hypothesis of impairments in fear and sadness recognition in CD participants high in psychopathic traits, we examined the relationship between YPI score and expression recognition by splitting the CD sample using the YPI cutoff of 2.5. Figure 2 shows facial expression recognition accuracy in high and lower psychopathy CD subgroups.

Comparing these subgroups revealed deficits in recognition of sadness (*Z* = −2.96, *p* = .003, *r* = .53), but no other emotions, in those high in psychopathic traits. This effect was not accounted for by subgroup differences in IQ (*p* = .48) or Benton Facial Recognition Test performance (*p* = .60). Repeating these analyses using callous-unemotional traits yielded similar results for sadness (*Z* = −2.17, *p* = .03, *r* = .41).

Only one control scored above the YPI cutoff, preventing us from using the above method to investigate effects of psychopathic traits on facial expression recognition in control subjects. However, neither overall psychopathic nor callous-unemotional traits were significantly correlated with sadness or fear recognition in control subjects using Spearman's rho.

### Aversive Conditioning

Data were unavailable for eight participants due to technical or experimenter error or inability to tolerate the procedure. Recall score did not differ between groups (*p* = .95); both could articulate the experimental contingencies.

### Autonomic Measures

The SCR data were not normally distributed, so they were normalized using a log(SCR + 1) transformation. Raw values are shown in the figures for ease of interpretation.

**Figure 3:**
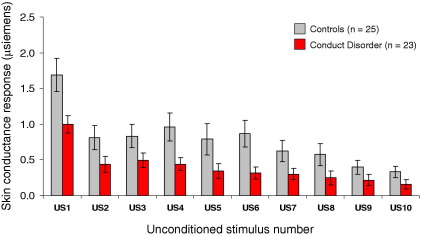
Skin conductance responses (SCRs) to the 10 presentations of the aversive unconditioned stimulus, by group. Although both groups showed marked habituation of SCRs to the unconditioned stimulus over time, control subjects showed significantly larger SCRs than conduct disorder participants across all trials. US, unconditioned stimulus.

We first examined whether SCRs to the US differed over time and by group, using repeated-measures analysis of variance (ANOVA) with time as within-subjects factor and group as between-subjects factor (10 × 2). This revealed effects of time [*F*(4.61,212.03) = 26.38, *p* < .001, η_p_^2^ = .37] and group [*F*(1,46) = 5.18, *p* < .05, η_p_^2^ = .10] but no interaction (*F* < 1). Skin conductance responses to the US were lower in CD participants than control subjects (Figure 3). Additionally, SCRs to the US declined strongly over time in both groups.

Group differences in autonomic conditioning were assessed using a mixed-model ANOVA with group (control, CD) as a between-subjects factor and CS type (CS+, CS−) and conditioning phase (HAB, ACQ1, ACQ2, EXT) as within-subjects factors (2 × 2 × 4). This revealed effects of CS type [*F*(1,46) = 21.01, *p* < .001, η_p_^2^ = .31] and conditioning phase [*F*(3,138) = 5.78, *p* < .005, η_p_^2^ = .11] but not group [*F*(1,46) = 3.11, *p* = .08]. Underlying the phase effect, SCRs were larger during HAB than ACQ1 or ACQ2 (both *p* ≤ .01). Underlying the CS type effect, subjects showed larger SCRs to the CS+ than the CS– (*p* < .001).

**Figure 4:**
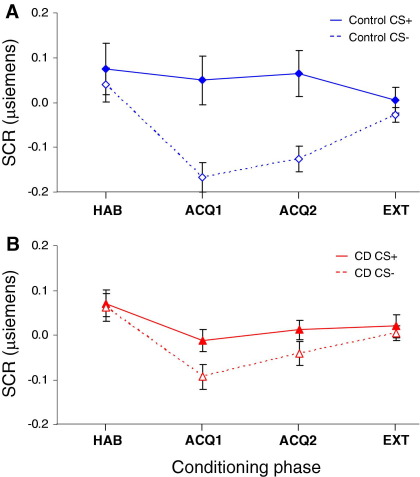
Mean (± SE) skin conductance responses to blue test slides (conditioned stimulus positive unpaired with unconditioned stimulus, solid line and closed symbols) and red control slides (conditioned stimulus negative, dashed line and open symbols) across conditioning phases in: **(A)** control and **(B)** conduct disorder groups. Control participants showed enhanced differential conditioning, as shown by a larger difference between skin conductance responses to the conditioned stimulus positive and the conditioned stimulus negative during acquisition phase 1 and acquisition phase 1, relative to conduct participants. ACQ1, acquisition phase 1; ACQ2, acquisition phase 2; CD, conduct disorder; CS–, conditioned stimulus negative; CS+, conditioned stimulus positive; EXT, extinction phase; HAB, habituation phase; SCR, skin conductance response.

We also observed significant group × CS type [*F*(1,46) = 3.98, *p* = .05, η_p_^2^ = .08] and conditioning phase × CS type [*F*(1.47,67.76) = 4.10, *p* < .05, η_p_^2^ = .08] interactions. Breaking down the former, control subjects exhibited greater SCR differentiation between CS types than CD participants (Figure 4), as shown using separate repeated-measures ANOVAs for each group, which revealed an effect of CS type in control subjects [*F*(1,24) = 31.45, *p* < .001, η_p_^2^ = .57] but not CD participants [*F*(1,22) = 2.46, *p* = .13].

The CS type × phase interaction was driven by a divergent pattern of SCRs to the respective CS types between habituation and acquisition 1 and 2. That is, the difference in SCRs between the CS+ and CS– was much greater during acquisition 1 and 2, relative to habituation. There was, however, no significant group × CS type × phase interaction.

To ensure that group differences in autonomic conditioning were not explained by IQ differences, we ran an analysis of covariance (ANCOVA) with IQ as a covariate. The IQ was a significant covariate, but after accounting for IQ, all results except the effect of CS type remained significant and a main effect of group emerged [*F*(1,45) = 12.76, *p* < .001, η_p_^2^ = .22]. To examine the impact of comorbidity, we excluded participants with ADHD and reran the analyses. The group × CS type interaction was not significant but had a similar effect size [*F*(1,41) = 3.42, *p* = .07, η_p_^2^ = .08]. Crucially, there was still no effect of CS type in this noncomorbid CD subgroup [*F*(1,17) = 1.52, *p* = .23]. The group × CS type interaction was significant [*F*(1,44) = 4.24, *p* < .05, η_p_^2^ = .09] following exclusion of participants with MDD.

To investigate the possibility that reduced US responsiveness caused the conditioning impairments, we excluded seven CD participants with small SCRs to the US (mean values < .2 μS across 10 US trials). There was no effect of CS type in the CD subgroup with normal SCRs to the US [*F*(1,15) = 2.0, *p* = .17].

In separate ANCOVAs controlling for tobacco, alcohol, or cannabis use, the group × CS type interaction remained significant in each case (all *p* < .05, η_p_^2^ ≥ .09). Finally, we explored whether psychopathic traits influenced fear conditioning. In an ANCOVA with psychopathic traits as a covariate, this variable was not a significant covariate when considering the entire sample. We also assessed for differences in fear conditioning between the low and high psychopathy CD subgroups (Figure S2 in Supplement 1). There was no effect of subgroup status (*p* = .73) nor a subgroup × CS type interaction (*p* = .59). Neither subgroup showed an effect of CS type (both *p* > .12).

### Startle Reflex Modulation by Affective Valence

Data were unavailable for seven participants due to technical or experimenter error or parents withholding consent for their children to participate in this experiment.

**Figure 5:**
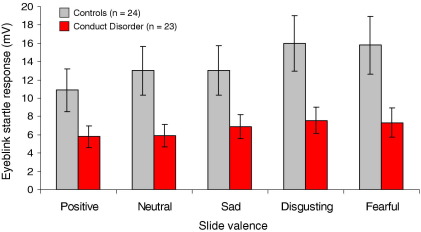
Mean (± SE) startle reflex magnitudes to a 97-dB acoustic probe when viewing pictures of different affective valence, by group. Compared with control subjects, conduct disorder participants exhibited reduced startle magnitudes (*p* < .05) regardless of slide valence, although the pattern of affective modulation did not differ significantly between groups. mV, millivolt.

The startle data were not normally distributed, so they were normalized using a square root transformation. Absolute values are provided in Figure 5 for ease of interpretation.

We ran a repeated-measures ANOVA with slide category as a within-subjects factor and group as a between-subjects factor (5 × 2). This revealed effects of slide category [*F*(4,180) = 14.79, *p* < .001, η_p_^2^ = .25] and group [*F*(1,45) = 4.61, *p* < .05, η_p_^2^ = .09] but no interaction effect (*p* = .09). Pairwise comparisons between slide categories showed reduced startle magnitudes to the acoustic probe when participants viewed positive slides, relative to all negative slides (all *p* < .005). Startle magnitudes were larger when viewing disgusting and fearful slides compared with neutral slides (both *p* < .005) and when viewing disgusting compared with sad slides (*p* < .005). Thus, startle magnitudes were significantly modulated by slide category.

Underlying the group effect, CD participants exhibited lower startle magnitudes across all slide categories (Figure 5). This effect remained significant (*p* < .05, η_p_^2^ = .15) following exclusion of participants with average startle responses <2.5 mV (nonresponders to the startle probe [31]) and following statistical adjustment for differences in IQ and psychopathic traits (both *p* < .05). Furthermore, it was marginally significant following exclusion of participants with MDD (*p* = .06, η_p_^2^ = .08) or ADHD (*p* = .06, η_p_^2^ = .08).

The influence of psychopathy on affective modulation was explored further by splitting the CD sample into high and low psychopathy subgroups. There was no subgroup effect on overall startle magnitudes (*p* = .88) or a psychopathy subgroup × slide category interaction (*p* = .42; Figure S3 in Supplement 1). Hence, there was no evidence for reduced affective modulation in the high relative to the low psychopathy CD subgroup.

Finally, group effects on startle magnitude remained significant when controlling for differences in tobacco (*p* < .05, η_p_^2^ = .11), cannabis (*p* = .05, η_p_^2^ = .08), or alcohol use (*p* < .005, η_p_^2^ = .18).

## Discussion

The objective of this study was to investigate emotional recognition, learning, and reactivity in female adolescents with CD. Consistent with predictions, female subjects with CD showed impaired recognition of anger and disgust relative to control subjects. This pattern was similar to that previously observed in male subjects with early-onset CD (14) and adults with impulsive aggression problems (52). Although impaired anger recognition in CD may be considered counterintuitive given evidence for hostile biases in social information processing in aggressive individuals (53), our data provide further evidence for reduced, rather than increased, sensitivity to hostile facial signals in CD. This observation is important because facial expressions convey information about the feelings and intentions of others. In particular, angry expressions can be considered social punishment signals, informing the observer that they should stop their current behavioral action (54).

In addition to showing that female subjects with CD scored higher than control subjects on measures of psychopathic and callous-unemotional traits, we demonstrated that variation in these personality traits influenced recognition of sad expressions. However, contrary to predictions, variation in psychopathic traits did not affect fear recognition. Several studies have demonstrated impaired fear and sadness recognition in children (15,55) and adults (56–58) with psychopathic traits, although conflicting findings are reported (59,60).

The present findings on startle reflex modulation do not provide clear evidence for aberrant modulation by affective valence in female subjects with CD. Earlier research in boys with CD or ODD also did not reveal an abnormal pattern of affective modulation (13,33). These data indicate that physiological sensitivity to the emotional valence of visual stimuli is intact in some individuals with CD, and as a group they do not show an aberrant pattern of affective modulation as observed in adult psychopaths (30–32,61). Furthermore, splitting the CD sample into those high and low in psychopathic traits did not reveal deficient affective modulation in the former. The CD participants in this study did, however, show reduced startle responses to the acoustic probe used to elicit the startle reflex, regardless of slide affective valence, consistent with previous results (13,33).

Further evidence for reduced autonomic responsiveness in female subjects with CD comes from our aversive conditioning experiment. Although both groups displayed strong SCR habituation to the aversive US, responses to this stimulus were consistently lower in participants with CD than control subjects. With respect to autonomic conditioning to neutral stimuli, we observed a group × CS type interaction, underpinned by control subjects showing increased SCRs to the CS+ (which predicted the US) relative to the CS– (which did not predict the US), whereas CD participants failed to differentiate between these stimuli. Female subjects with CD therefore showed impaired fear conditioning, consistent with previous data from male adolescents with CD (13) and adult psychopaths (18–21). Longitudinal studies have shown that impaired fear conditioning in early childhood may predict involvement in criminal activity by age 23 (62), whereas enhanced conditioning may act as a protective factor (63).

### Theoretical Implications

Our results, demonstrating deficits in emotional recognition and learning in CD, may help to explain why individuals with CD experience interpersonal difficulties and problems in learning from punishment. These impairments did not appear to be explained by lower cognitive ability (a general deficit) in the CD group, because all results remained significant following adjustment for IQ differences or exclusion of high IQ control subjects. Additionally, our nonaffective control tasks (assessing facial identity recognition and awareness of the CS+-US contingency in the conditioning experiment) showed no group differences, providing further evidence for a disproportionate impairment in emotional functioning in CD. Because conditioning deficits and reduced startle magnitudes are reported in patients with amygdala lesions (64–67), our findings may be interpreted as evidence for amygdala dysfunction in CD. However, the lack of deficits in fear recognition or affective modulation in female subjects with CD is problematic for this position because amygdala lesions are associated with impairments in these processes (68–70). Further research using neuroimaging techniques is required to investigate the neural basis of the behavioral and autonomic differences reported in this study.

Our results provide indirect support for the delayed-onset pathway model of antisocial behavior in female subjects (71). This proposes that female subjects with adolescence-onset CD should show neuropsychological vulnerabilities in common with male subjects with early-onset CD, even though the former show a delay in the emergence of their clinical phenotype. A key question for future research is why, despite the presence of these neuropsychological vulnerabilities, female subjects with CD generally do not exhibit severe antisocial behavior until adolescence (1,72).

Five limitations are noted. First, the sample was relatively small, which may have made it difficult to demonstrate aberrant affective modulation of startle due to power limitations. Second, because we had to analyze the facial expression recognition data using nonparametric statistics, we were unable to control for group differences in IQ using ANCOVA procedures. We instead equated groups for IQ by omitting high IQ control subjects but cannot exclude the possibility that group differences in facial expression recognition were partially driven by IQ. Third, conflating early-onset and adolescence-onset forms of CD (plus CD and ODD) was not optimal. These findings thus require replication in a larger sample, ideally with a prospective design to avoid using retrospective reports of symptom onset (the fourth limitation). Fifth, using a similar aversive noise as the US in our conditioning task and as the acoustic probe in our startle experiment was not ideal.

### Conclusions

This study provides evidence for deficits in emotional recognition and learning in female subjects with CD, consistent with previous results in male subjects with early-onset CD. Our findings therefore suggest that neural systems involved in emotional recognition and learning may be compromised in CD. We found no clear evidence for reduced emotional reactivity but instead observed a generalized deficit in autonomic reactivity in female subjects with CD.

## Figures and Tables

**Table 1:** Participant Characteristics

	Control Subjects (*n* = 30)	CD (*n* = 25)	*p* Value
Age (years, mean ± SD)	15.3 ± .7	15.6 ± 1.0	.279
Estimated IQ (mean ± SD)	108.3 ± 14.0	97.2 ± 11.0	.002
MDD Diagnosis	0^a^	2	
ADHD Diagnosis	0^a^	5	
Psychopathic Traits (YPI)	1.94 ± .35	2.44 ± .35	<.001
Anxiety	11.5 ± 6.9	13.0 ± 6.3	.396
SES			
Low	5	4	
Middle	10	0	.799
High	15	10	
Ethnicity			
Caucasian	29	23	.448
Nonwhite	1	2	
Regular Use of:			
Tobacco	3 (10%)	20 (80%)	<.001
Alcohol	11 (37%)	19 (76%)	.002
Cannabis	3 (10%)	15 (60%)	<.001

Socioeconomic status information was unavailable for one conduct disorder participant. ADHD, attention-deficit/hyperactivity disorder; CD, conduct disorder; MDD, major depressive disorder; SES, socioeconomic status; YPI, Youth Psychopathic traits Inventory.

a The presence of major depressive disorder or attention-deficit/hyperactivity disorder was an exclusion criterion for the control group.
