# Supplementary material for: Facial Expression Recognition, Fear Conditioning, and Startle Modulation in Female Subjects with Conduct Disorder
Source: Biol Psychiatry. 2010 Aug 1;68(3):272–9. doi: 10.1016/j.biopsych.2010.02.019 (PMC2954286; doi:10.1016/j.biopsych.2010.02.019)
Supplement: Supplement 1 [file mmc1.pdf]

Supplemental Information

Table S1A. Confusion matrix data for the healthy control group

	**Actual expression depicted**					
Identified as:	Anger	Disgust	Fear	Happiness	Sadness	Surprise
**Anger**	**78.3**	5.7	0.0	0.8	0.3	0.0
**Disgust**	16.2	**84.0**	1.5	0.0	3.7	0.7
**Fear**	2.2	0.5	**83.7**	0.8	2.8	7.3
**Happiness**	0.3	0.2	0.3	**97.3**	0.0	1.2
**Sadness**	0.5	9.3	0.2	0.7	**93.2**	0.0
**Surprise**	2.5	0.3	14.3	0.3	0.0	**90.8**

Table S1B. Confusion matrix data for the Conduct Disorder group

	**Actual expression depicted**					
Identified as:	Anger	Disgust	Fear	Happiness	Sadness	Surprise
**Anger**	**60.6**	19.2	0.8	0.4	0.2	0.6
**Disgust**	25.8	**62.6**	1.2	0.0	2.2	1.2
**Fear**	5.4	0.4	**76.8**	0.2	3.0	11.0
**Happiness**	0.0	0.2	0.0	**97.8**	0.4	1.2
**Sadness**	1.2	17.0	0.4	1.2	**93.8**	0.4
**Surprise**	7.0	0.6	20.8	0.4	0.4	**85.6**

The above tables show confusion matrices for the control (Table S1A) and Conduct Disorder (Table S1B) groups. In each case, the facial expression depicted is displayed in the columns, and the group's choice of emotion labels in the rows. Percent correct recognition of the relevant emotion is shown in bold.

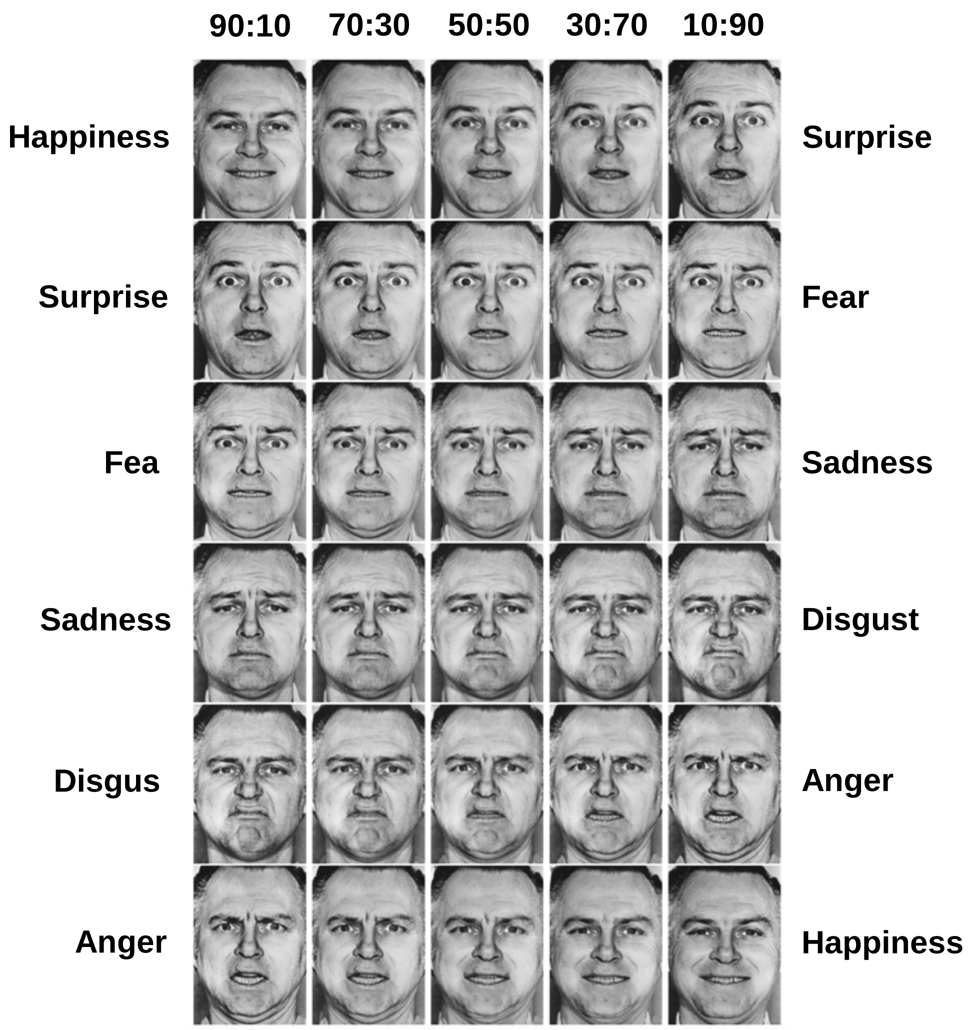

Figure S1. Facial expression continua used in the Emotion Hexagon task. Running from left to right, the columns show 90%:10%, 70%:30%, 50%:50%, 30%:70% and 10%:90% morphs along each continuum. From top to bottom, the continua shown in each row are Happiness-Surprise, Surprise-Fear, Fear-Sadness, Sadness-Disgust, Disgust-Anger, and Anger-Happiness, respectively.

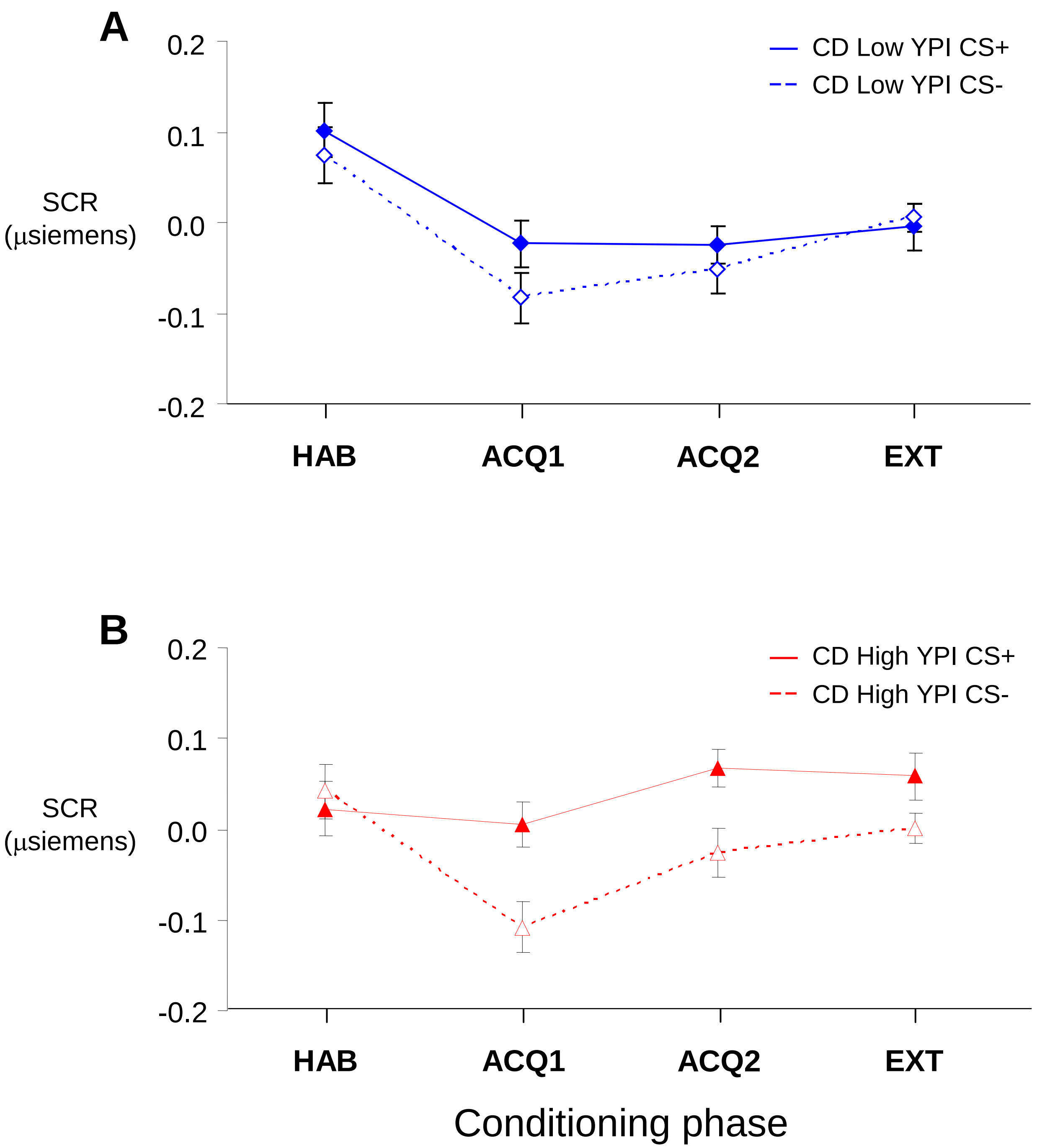

Figure S2. Mean (± SE) skin conductance responses (SCRs) to blue test slides (CS+ unpaired with US, solid line and closed symbols) and red control slides (CS-, dashed line and open symbols) across conditioning phases, in: (**A**) CD participants with lower levels of psychopathic traits, and (**B**) CD participants with high levels of psychopathic traits. Neither subgroup showed a significant effect of CS type (both $p > .12$). ACQ1, acquisition phase 1; ACQ2, acquisition phase 2; CD, conduct disorder; CS, conditioned stimulus; EXT, extinction phase; HAB, habituation phase; US, unconditioned stimulus; YPI, Youth Psychopathic Traits Inventory.

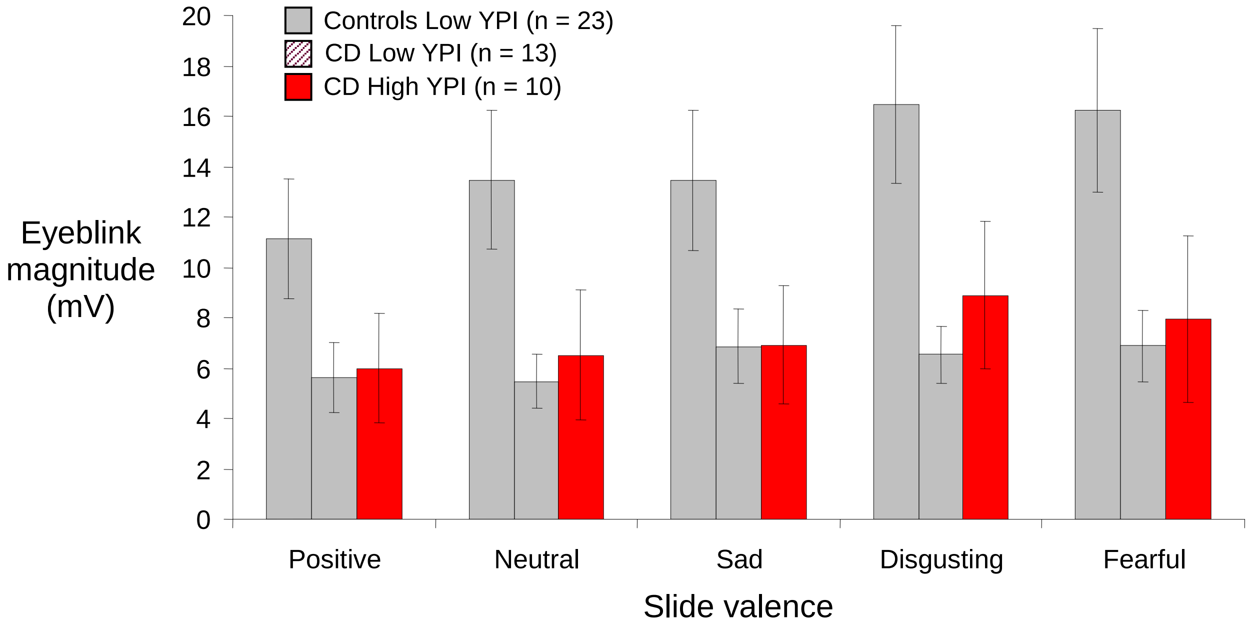

Figure S3. Mean (± SE) startle reflex magnitudes to a 97dB acoustic probe when viewing pictures of different affective valence. The CD group is subdivided into those with either low or high levels of psychopathic traits (as measured using the Youth Psychopathic Traits Inventory). This shows that controls low in psychopathic traits had larger startle magnitudes than both CD subgroups. It also illustrates that modulation of the startle reflex by slide category was most attenuated in the *low* psychopathy CD subgroup, although there was no main effect of subgroup status or significant interaction between psychopathy subgroup and slide category. CD, Conduct Disorder; YPI, Youth Psychopathic Traits Inventory
